# Apatinib inhibits tumour progression and promotes antitumour efficacy of cytotoxic drugs in oesophageal squamous cell carcinoma

**DOI:** 10.1111/jcmm.17209

**Published:** 2022-03-21

**Authors:** Yanyan Chi, Feng Wang, Yana Zhang, Zhengzheng Shan, Weili Tao, Yujin Lian, Dao Xin, Qingxia Fan, Yan Sun

**Affiliations:** ^1^ Department of Oncology The First Affiliated Hospital of Zhengzhou University Zhengzhou China

**Keywords:** Akt/mTOR, apatinib, cytotoxic drugs, oesophageal cancer

## Abstract

Apatinib, a highly selective inhibitor of vascular endothelial growth factor receptor‐2 (VEGFR‐2), inhibits the angiogenesis of tumours. The function and mechanism of apatinib in oesophageal squamous cell carcinoma (ESCC) remain unknown. In present study, we found that the development of ESCC in patients was controlled by treatment of combination of apatinib and a chemotherapeutic drug. Moreover, apatinib efficiently promotes cell apoptosis, inhibits cell proliferation, invasion, epithelial‐mesenchymal transition (EMT) and activity of the Akt/mTOR pathway in ESCC cells. Western blot analysis showed that apatinib significantly increased vimentin protein levels, decreased Bcl2, matrix metalloproteinase 9 (MMP9), E‐cadherin, p‐Akt and p‐mTOR protein levels in ESCC cells. Furthermore, apatinib enhanced chemosensitivity of cytotoxic drugs paclitaxel (TAX), 5‐fluorouracil (5‐FU) and cisplatin (DDP) by upregulating expression of vimentin protein, and downregulating expression of Bcl2, MMP9 and E‐cadherin protein in vitro. Compared with single‐agent groups, the combination of apatinib with each chemotherapeutic drug significantly repressed tumour growth and angiogenesis through blocking the expression of Ki67 and VEGFR‐2 in vivo. Taken together, apatinib efficiently inhibits cell growth through blocking Bcl2 and Akt/mTOR pathway, and suppresses metastasis via inhibiting MMP9 and EMT in ESCC cells. Apatinib promoted antitumour effect of chemotherapeutic agents through promoting cell apoptosis and inhibiting EMT and angiogenesis in ESCC.

## 1 INTRODUCTION

Oesophageal carcinoma (ESCA) is one of the most common digestive tract malignant tumours in the world.^1^ Oesophageal squamous cell carcinomas (ESCC) is the main pathological type in China, especially in Henan province where there is a higher incidence. ESCC in its early stages often affects its functions only slightly, with no obvious symptoms. When they have clinical manifestations, most of the patients are in the advanced stage and have missed the chance of surgical treatment. Chemotherapy is an essential method to advanced ESCC. However, the clinical efficacy of chemotherapy was limited, and the overall survival (OS) of patients with first‐line treatment is only 5–10 months. Recurrence, metastasis and resistance to current chemotherapy are still the primary causes of death in ESCC patients.

Apatinib is a small molecule tyrosine kinase inhibitor of VEGFR‐2.^2^ It highly selective competes for the ATP binding site of VEGFR‐2 so that blocks downstream signal transduction and inhibits the production of tyrosine kinase, which inhibits the process of tumour angiogenesis.^3^ It has been reported that apatinib has antitumoral effect on a few malignancies.^4^, ^5^, ^6^, ^7^ Apatinib prolongs median overall survival (mOS) and progression‐free survival (mPFS) of patients with advanced gastric cancer.^4^ Lower daily doses of apatinib have greater benefits than higher daily doses in advanced gastric cancer patients.^8^ Apatinib also has reliable efficacy in advanced non‐small‐cell lung cancer (NSCLC) patients and chemotherapy‐refractory metastatic colorectal cancer.^6^, ^9^ Furthermore, apatinib enhances antitumour effect of chemotherapeutic drugs on several cancers.^10^, ^11^, ^12^, ^13^ Qin et al. found the synergistic effect between apatinib and the combination of the chemotherapy drugs TAX, oxaliplatin (L‐OHP), 5‐FU in gastric cancer.^14^ Another study confirmed that apatinib inhibited tumour growth in cervical cancer and synergizes with TAX.^15^ Moreover, it is reported that the combination of apatinib and docetaxel may prolong survival of patients with advanced oesophageal carcinoma.^16^ However, the mechanism of apatinib in oesophageal cancer and the mechanism of synergistic antitumoral effect of apatinib combing with cytotoxic drugs in oesophageal cancer are not clear.

Previous studies have revealed that Akt/mTOR signalling pathway plays an important role in progression of cancers. Akt/mTOR pathway regulates cell growth and metastasis of cancer cells, including ESCC cells. Additionally, apatinib promotes apoptosis and autophagy through inactivating STAT3 pathway, and inhibits invasion and EMT in a few cancer.^17^, ^18^, ^19^

In present study, we found that the development of ESCC in patients was controlled by treatment of combination of apatinib and a chemotherapeutic drug. Then, we explored the function and mechanism of apatinib in the treatment of oesophageal cancer. We assessed the effect of cytotoxic drugs TAX, 5‐FU and DDP with or without apatinib on oesophageal cancer in vitro and in vivo. The results demonstrate that apatinib enhances antitumour efficacy of cytotoxic drugs through promoting cell apoptosis and inhibiting EMT in ESCC cells, suppresses tumour growth and angiogenesis via blocking Ki67 and VEGFR‐2.

## 2 MATERIALS AND METHODS

### 2.1 Patients and treatment

Two patients were enrolled in our study. Case 1 was diagnosed with ESCC and retroperitoneal lymph node metastasis, and case 2 was diagnosed with ESCC and mediastinal lymph node metastasis. They never received chemotherapy, radiotherapy or surgery. All of the protocols used in this study were approved by the Medical Research Ethics Committee of our Hospital. The informed consent has been signed.

Patients received treatment of Nedaplatin and Gimeraciland Oteracil Porassium Capsules. Nedaplatin 80 mg/m^2^ (intravenously guttae, ivgtt) was given on Day 1; Gimeraciland Oteracil Porassium capsules were given 60 mg (P.O.) twice a day in first 14 days. If new lesions occur, they were treated with the combination of apatinib and docetaxel. Apatinib was administered 500 mg (P.O.) once a day continuously; docetaxel 75 mg/m^2^ (ivgtt) was given on Day 1.

### 2.2 Bioinformatic analysis

The expression of VEGFR‐2 in patients with ESCA and normal was detected by bioinformatic algorithms from the publicly available databases TCGA and GEPIA. The cut‐off was defined; the overall survival (OS) of high level and low level of VEGFR‐2 group was predicted by GEPIA. The cut‐off value was determined; the overall survival (OS) of the high and low levels of VEGFR‐2 in ESCA patients was predicted by GEPIA.

### 2.3 Agents

Apatinib, 5‐FU, TAX and DDP were used in the present study. In vitro studies, the stock solution containing apatinib (Hengrui), 5‐FU (Sigma), TAX (Sigma) and DDP (Sigma) were prepared in dimethyl sulfoxide (DMSO). In vivo assays, apatinib was dissolved in DMSO and diluted in physiological saline. 5‐FU, TAX and DDP injection were diluted in physiological saline.

### 2.4 Cell culture

Oesophageal squamous cell carcinomas cell lines (KYSE450, EC1) were preserved in our laboratory. They were cultured in RMPI 1640 medium (Hyclone) supplemented with 10% foetal bovine serum (Hyclone) and 1% penicillin–streptomycin at 37°C with 5% carbon dioxide. The cells were passed on every other day, and the cells of logarithmic growth phase were collected for subsequent experiments.

### 2.5 Proliferation assay

KYSE450 cells were maintained in 96‐well plates (5000 cells/well) and treated with 0, 10, 20, 30, 40 and 60 µmol/L apatinib, 0, 2.5, 5, 10, 50 and 125 µmol/L 5‐FU, 0, 1.5625, 3.125, 6.25, 12.5 and 25 nmol/L TAX, 0, 1, 2, 4, 8 and 16 µmol/L DDP. EC1 cells were maintained in 96‐well plates (2000 cells/well) and treated with 0, 10, 20, 30, 40 and 60 µmol/L apatinib, 0, 10, 50, 200, 400 and 800 µmol/L 5‐FU, 0, 1.5625, 3.125, 6.25, 12.5 and 25 nmol/L TAX, 0, 1, 2, 4, 8 and 16 µmol/L DDP. Cell viability was determined by CCK‐8 assay after 24, 48 and 72 h.

Then, the experiment is divided into 8 groups: control group (DMSO), 5‐FU group, TAX group, DDP group, apatinib group, Apatinib + 5‐FU group, apatinib + TAX group and Apatinib + DDP group. ESCC cells (KYSE450 and EC1) were maintained in 96‐well plates (5000 cells/well) and treated with agents. After 48 h, cell viability was determined by CCK‐8 assay.

### 2.6 Cell apoptosis assay

Flow cytometry was performed to detect cell apoptosis using an Annexin V‐FITC/PI apoptosis detection kit (BD Biosciences) on the basis of instructions. Cells were planted in 6‐well plate and treated with agents. We harvested the treated cells (KYSE450 and EC1) and cleaned them with cold PBS twice. The cells were centrifuged at 2000 rpm for 5 min and resuspended at the density of 1 × 10^6^ /ml. Then, we stained them with Annexin V and PI in the dark, and analysed the data via flow cytometer.

### 2.7 Western blotting

Protein was extracted from oesophageal cancer cells using RIPA lysis buffer supplemented with protease inhibitor and phosphatase inhibitor. Then, separating total proteins was performed using gel electrophoresis, and transferring proteins to a polyvinylidene fluoride membrane (PVDF; Beyotime) was performed using electroblotting. The membranes were immersed in 5% skim milk in TBST for 1 h and incubated in the following primary antibodies: rabbit anti‐Bcl2 (1:1000; CST), E‐cadherin (1:5000; Proteintech), vimentin (1:1000; Proteintech), and MMP9 (1:1000; Proteintech), Akt and phosphorylated‐Akt (phosphoresce on S473, 1:5000; Abcam), S6 (1:1000, CST) and phosphorylated‐S6 (phosphoresce on S235/236, 1:2000, CST), rabbit anti‐GAPDH (1:4000; Proteintech). The membranes were washed by TBST and then incubated in secondary antibodies goat anti‐rabbit IgG (1:8000; Proteintech) for 1 h. ECL detection reagent (Santa Cruz) was dropped on the membranes in the dark.

### 2.8 Wound‐healing assay

Cell migration ability was measured by a wound‐healing assay. Cells were planted in 6‐well plate and treated with agents. We cultivated cells (KYSE450 and EC1) in 6‐well plates until their density become 80% conflux. Then, cells were wounded with an aseptic 10 µl plastic pipette tip and treated with agents. Then, images were captured before and after culturing for 48 h.

### 2.9 Transwell invasion assays

Cell invasive ability was measured by a 24‐well transwell plate (Corning). Transwell chamber was placed in a 24‐well plate and added with matrigel in a volumetric dilution of 1: 6. Cells (KYSE450 and EC1) were resuspended with serum‐free medium contained agents and moved them into the upper chamber, added 600 µl culture medium with 20% FBS to the lower chamber. Cells were incubated at 37°C for 24 h, fixed with 4% paraformaldehyde for 20 min, washed with PBS and then stained with 1% crystal violet for 30 min. Non‐migrated cells on the upper surface of the film were erased by cotton swabs, and migrated cells on lower surface of the film were counted.

### 2.10 Mouse xenograft model

EC1 cells (1 × 10^7^/100 μl/site) were injected into the right back side flanks of 5‐week‐old female BALB/c nude mice, and tumours were allowed to grow to 100 mm^3^. Then, the mice were randomly divided into 8 groups (*n* = 6 per group): control group, 5‐FU group, TAX group, DDP group, apatinib group, 5‐Fu + apatinib group, TAX + apatinib group and DDP + apatinib group. Mice were administered apatinib alone by oral gavage; 5‐FU, TAX and DDP by intraperitoneal injection; or apatinib in combination with each cytotoxic drug at the indicated dose and schedule (Table 1). Tumour volumes were measured every 3 days. After 14 days treatment, the tumours were removed and measured.

**TABLE 1:** In vivo efficacy of Apatinib in combination with cytotoxic drugs against ECSS tumour xenografts

Group	Agent, dose, route and schedule
Control	Vehicle i.g. qd × 14; Physiological saline i.p. q3d × 5
TAX	Vehicle i.g. qd × 14;10 mg/kg TAX i.p. q3d × 5
5‐FU	Vehicle i.g. qd × 14;30 mg/kg 5‐FU i.p. q3d × 5
DDP	Vehicle i.g. qd × 14;6 mg/kg DDP i.p. q3d × 5
Apatinib	Physiological saline i.p. q3d × 5; 75 mg/kg apatinib i.g. qd × 14
TAX + apatinib	10 mg/kg TAX i.p. q3d × 5; 75 mg/kg apatinib i.g. qd × 14
5‐FU + apatinib	30 mg/kg 5‐FU i.p. q3d × 5; 75 mg/kg apatinib i.g. qd × 14
DDP + apatinib	6 mg/kg DDP i.p. q3d × 5; 75 mg/kg apatinib i.g. qd × 14

Note

*n* = 6 (per group).

Abbreviations: i.g. intragastric administration; i.p., intraperitoneal injection.

### 2.11 Tunel

Sections were dewaxed in xylene for 5–10 min and then dewaxed in fresh xylene for 5–10 min. Sections were placed in absolute ethanol for 5 min, 90% ethanol for 2 min, 70% ethanol for 2 min and distilled water for 2 min. 2% DNase‐free proteinase K was added into tissues and incubated at 37°C for 20 min, then washed them with PBS three times. The samples were blocked with 5% serum at 37°C for 20 min. Prepared Tunel test solution and treated samples according to the instruction of In Situ Cell Death Detection Kit (Roche Applied Science). Images were acquired under a light microscope, and the apoptotic bodies are brownish yellow. The following formula was used to evaluate positive staining in the sections: Apoptotic rate = The number of positive cells per view/Total number of cells per view × 100%.

### 2.12 Immunohistochemistry

Tumour tissues were paraffin‐embedded and sliced into 4‐μm thick sections. The sections were incubated in 4°C overnight in the following primary antibodies: VEGFR‐2 (1:1600; CST), Ki67 (1:200; Abcam). The next day, sections were incubated in biotin‐labelled secondary antibody for 30 min at room temperature and then incubated in horseradish peroxidase labelled streptavidin. DAB solution was used for showing positive reactions, and haematoxylin was used for counterstaining.

### 2.13 Statistical analysis

SPSS (version 21.0; SPSS, Inc.) was used to analyses the data. The data among the groups were detected by one‐way analysis of variance (ANOVA) and shown as the mean ± SEM. *p* < 0.05 was regarded as statistical significance.

## 3 RESULT

### 3.1 Combination of apatinib and chemotherapeutic drug has synergistic antitumour effects in patients with ESCC

**FIGURE 1:**
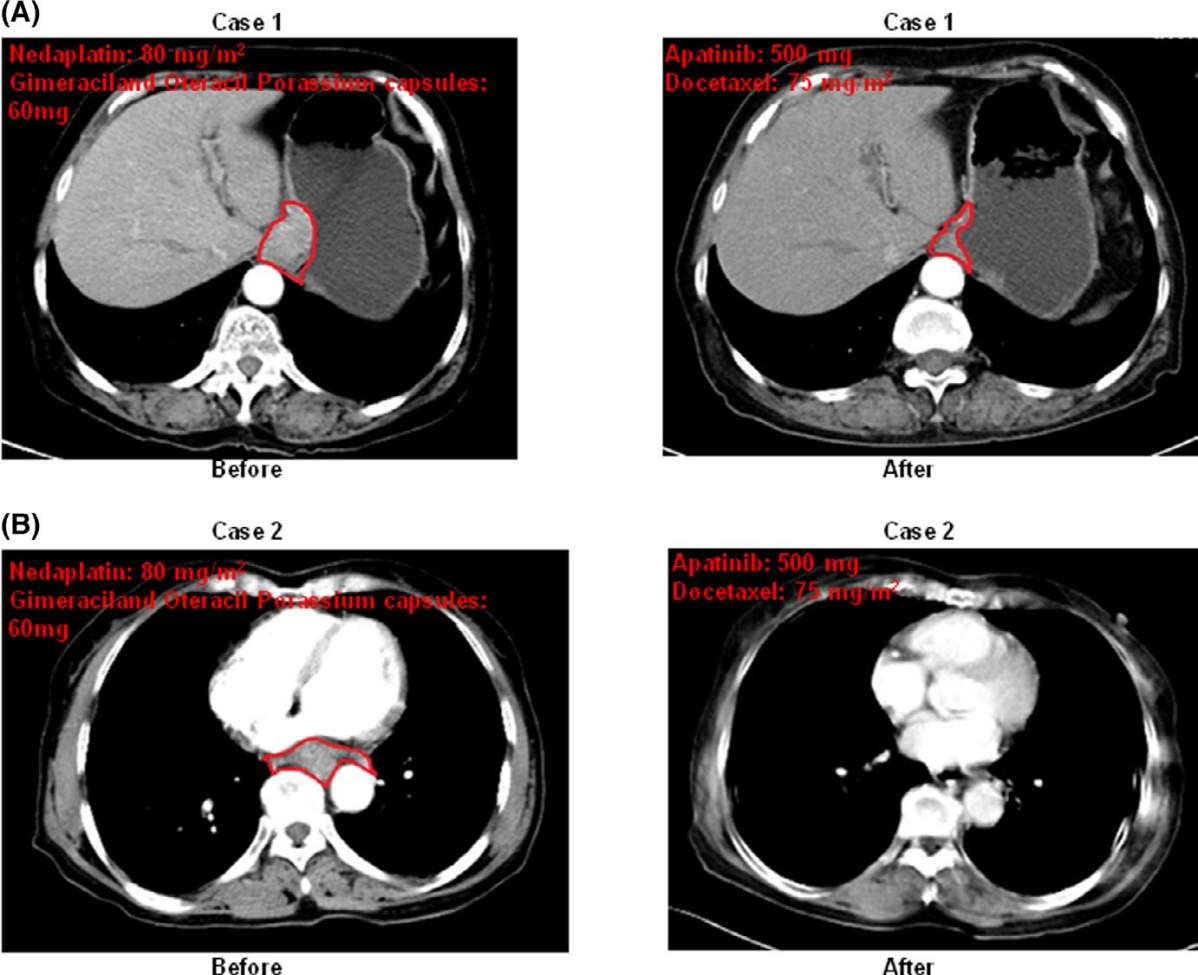
Computed tomography (CT) was used to detect the lesions before and after treatment with combination of apatinib and chemotherapeutic drug. The range of lesions were marked with red dotted lines (R, right; L, left). Case 1(A) was diagnosed with ESCC and retroperitoneal lymph node metastasis; case 2(B) was diagnosed with ESCC and mediastinal lymph node metastasis. Patients received treatment of Nedaplatin and Gimeraciland Oteracil Porassium Capsules. Nedaplatin 80 mg/m^2^ (intravenously guttae, ivgtt) was given on Day 1; Gimeraciland Oteracil Porassium capsules were given 60 mg (P.O.) twice a day in first 14 days. When the new lesions occurred, they were treated with the combination of apatinib and docetaxel. Apatinib was administered 500 mg (P.O.) once a day continuously; docetaxel 75 mg/m^2^ (ivgtt) was given on Day 1

Patient 1 received treatment of Nedaplatin and Gimeraciland Oteracil Porassium Capsules for four cycles. New lesions were found in the patient's stomach. Then, the combination of apatinib and docetaxel was used to treat the patient for six cycles. We found the range of stomach lesions was decreased. Patient 2 received treatment of Nedaplatin and Gimeraciland Oteracil Porassium Capsules for two cycles. We found new lesions in right lung of the patient. Then, the lower oesophageal lesion was reduced through the combination of apatinib and docetaxel for six cycles. Computed tomography (CT) was used to detect the lesions before and after treatment with combination of apatinib and chemotherapeutic drug (Figure 1).

### 3.2 The ESCA patients with high level of VEGFR‐2 have poor prognosis

**FIGURE 2:**
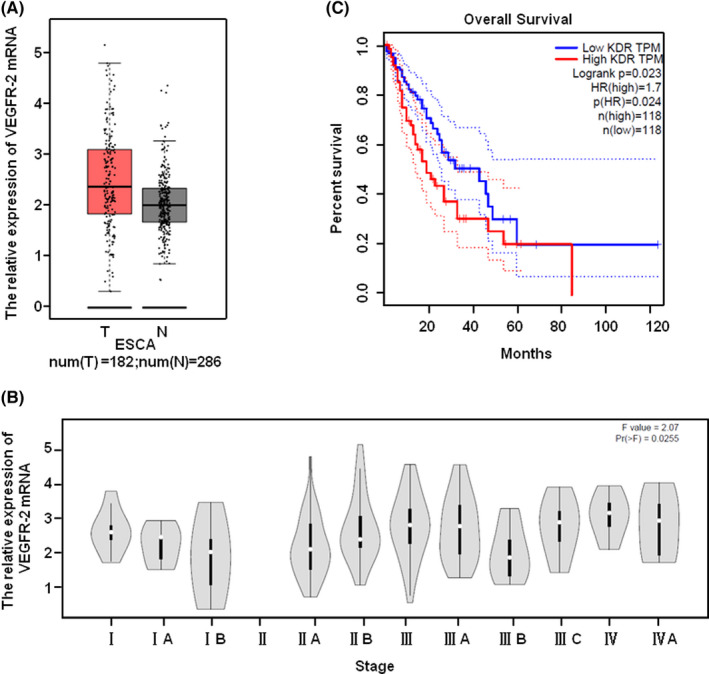
High expression of VEGFR‐2 in ESCC patients has poor prognosis. (A) The expression of VEGFR‐2 in ESCA patients and normal in TCGA database. (B) The expression of VEGFR‐2 in ESCA patients with different clinical stages in TCGA database. (C) The OS of ESCA patients with high VEGFR‐2 expression or low VEGFR‐2 expression in TCGA database

Apatinib is a small molecule tyrosine kinase inhibitor of VEGFR‐2. To explore the function and mechanism of apatinib in ESCC, we analysed the expression of VEGFR‐2 in the tissue database of ESCA patients. Although there is no statistical significance, we found that the levels of VEGFR‐2 were upregulated in ESCA patients compared with normal in TCGA database through the bioinformatic analysis (Figure 2A). Moreover, the VEGFR‐2 expression in stage Ⅲ and stage Ⅳ was higher than that in stage Ⅰ and stage Ⅱ (Figure 2B). After defining the cut‐off value, we analysed the OS of ESCA patients with VEGFR‐2 overexpression or downregulation. The results showed that the OS of ESCA patients with low level of VEGFR‐2 was longer than that of patients with high level of VEGFR‐2 (Figure 2C).

### 3.3 Apatinib inhibits cell proliferation and promotes cell apoptosis in ESCC cells

**FIGURE 3:**
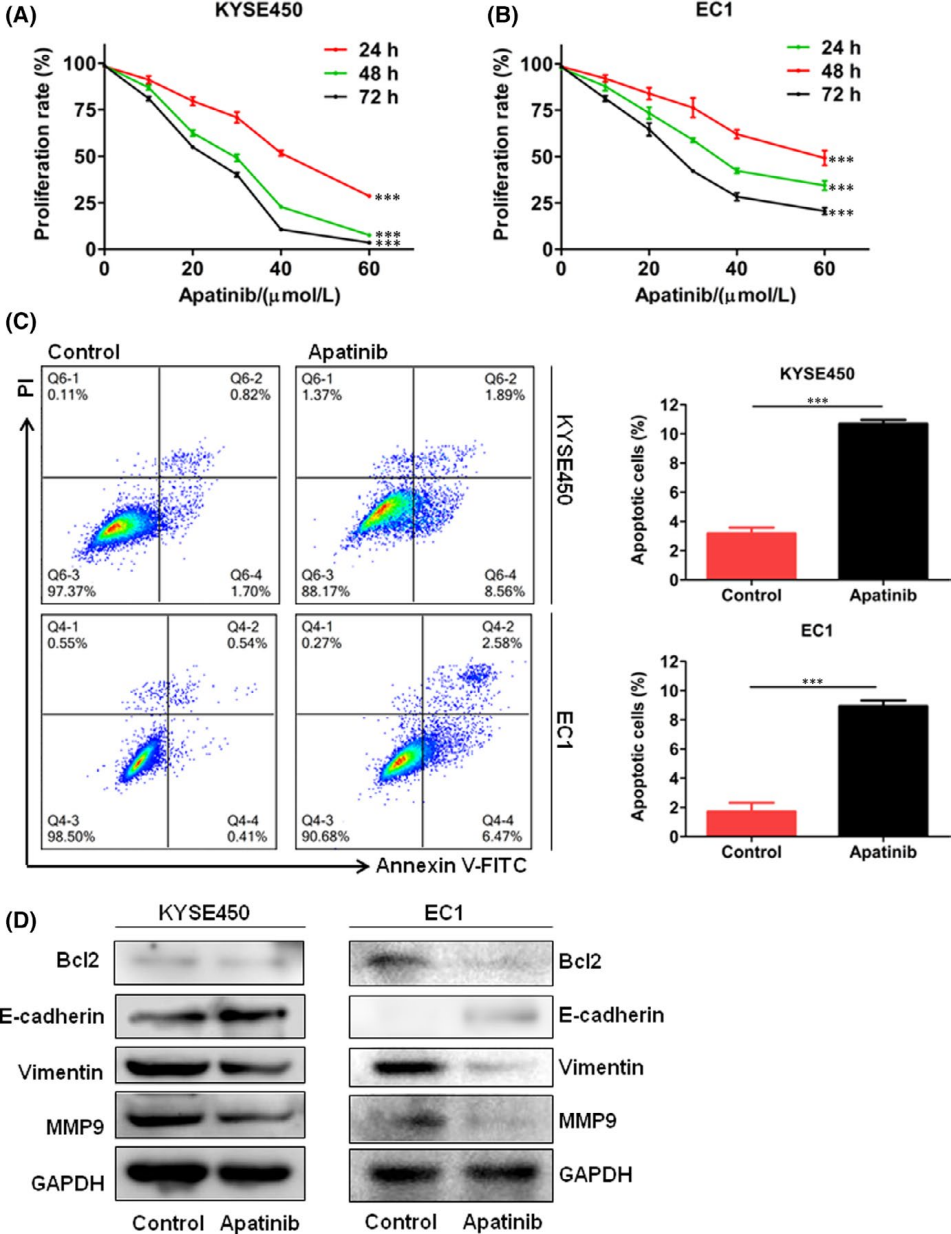
Effects of apatinib on ESCC cell growth in vitro. (A and B) KYSE450 and EC1 cells were treated with apatinib at the indicated concentration (10, 20, 30, 40 and 60 µmol/L) for 24, 48 and 72 h. Cell viability was determined by CCK‐8 assay. Cells treated with 0.1% DMSO was used as a control. (C) KYSE450 and EC1 cells were treated with apatinib at the indicated concentration for 48 h. Cell apoptosis rate was analysed in KYSE450 and EC1 cells. Cell apoptosis rates between different treatment groups were presented in percentages as histogram graphs. Cells treated with 0.1% DMSO were used as a control. (D) Western blot results of Bcl2, E‐cadherin, vimentin and MMP9 protein expression in KYSE450 and EC1 cells treated with apatinib. Data are shown as mean ± SEM (*** *p* < 0.001)

To explore the role of apatinib in ESCC cell proliferation, we treated KYSE450 and EC1 cells with apatinib in different concentrations. After 24, 48 and 72 h, cell viability of oesophageal cancer cells was determined by CCK‐8. We found that apatinib markedly inhibited cell proliferation in KYSE450 and EC1 cells compared with untreated control group with the increase of drug dose and treatment time (Figure 3A,B).

In order to explore the effects of apatinib on cell apoptosis of ESCC cells, we added apatinib into KYSE450 and EC1 cells and detected cell apoptosis by flow cytometry. The results showed that apatinib promoted apoptosis of KYSE450 and EC1 cells compared with control group (Figure 3C). To verify these results, we examined the changes of apoptotic related proteins in KYSE450 and EC1 cells treated with apatinib or DMSO (control group). Western blot analysis showed that apatinib significantly decreased Bcl2 protein levels in KYSE450 and EC1 cells (Figure 3D).

### 3.4 Apatinib inhibits cell migration, invasion and EMT in ESCC cells

**FIGURE 4:**
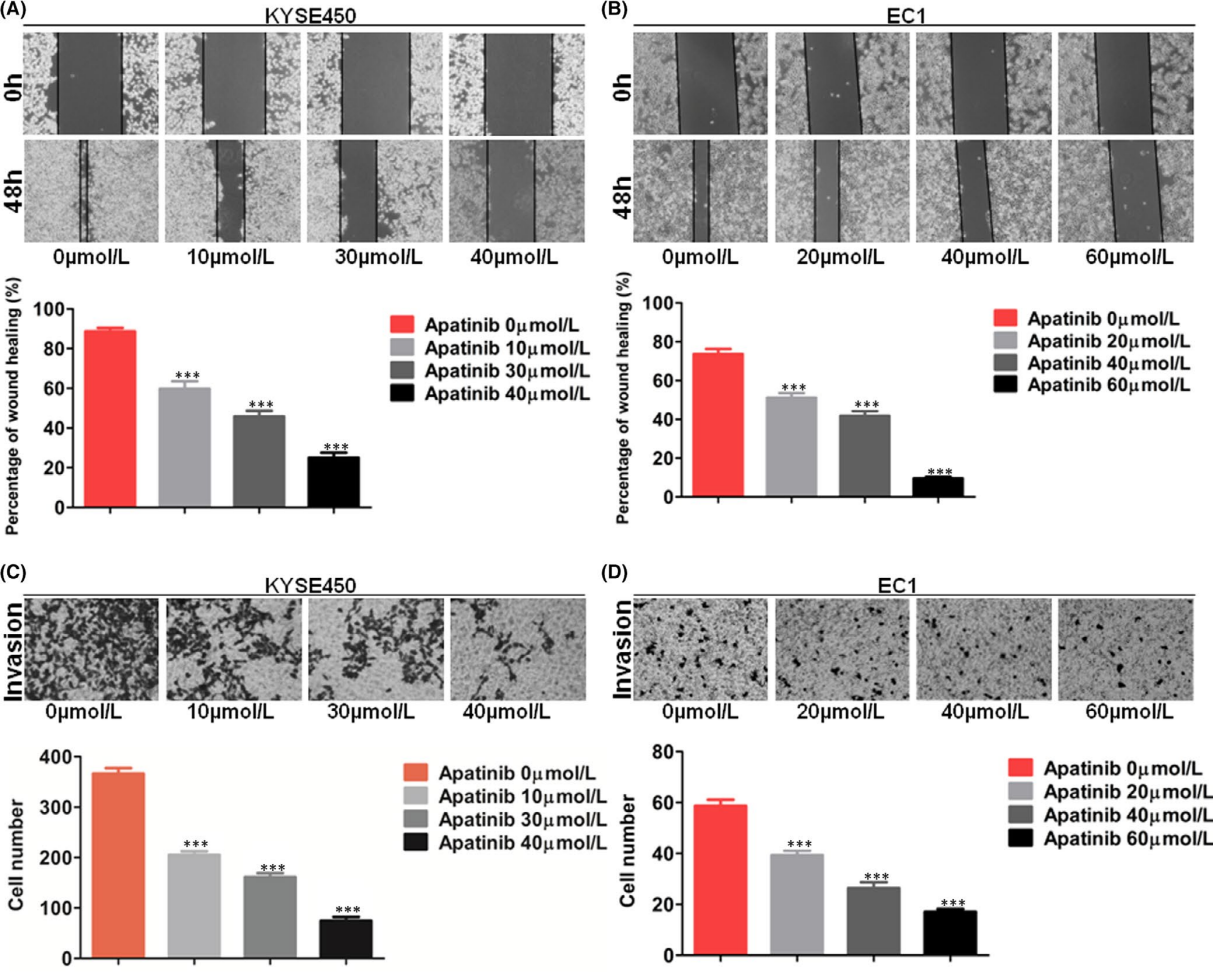
Apatinib inhibited migration and invasion of ESCC cells. (A and B) Wound‐healing assays showed that apatinib remarkably decreased KYSE450 and EC1 cells migration (×100). (C and D) Transwell assays showed that apatinib remarkably decreased KYSE450 and EC1 cells invasion (×400). Data are shown as mean ± SEM (*** *p* < 0.001)

KYSE450 and EC1 cells were wounded and treated with apatinib. We calculated the healing rates and found that apatinib inhibited migration of KYSE450 and EC1 cells (Figure 4A,B). Moreover, we detected invasion ability of KYSE450 and EC1 cells treated with apatinib or DMSO via transwell chamber assay. We found that the number of invasive cells treated with apatinib was less than control group (Figure 4C,D). Western blot analysis showed that apatinib decreased the levels of MMP9 and vimentin, and increased the levels of E‐cadherin in KYSE450 and EC1 cells (Figure 3D).

### 3.5 Apatinib reduces activity of the Akt/mTOR pathway

**FIGURE 5:**
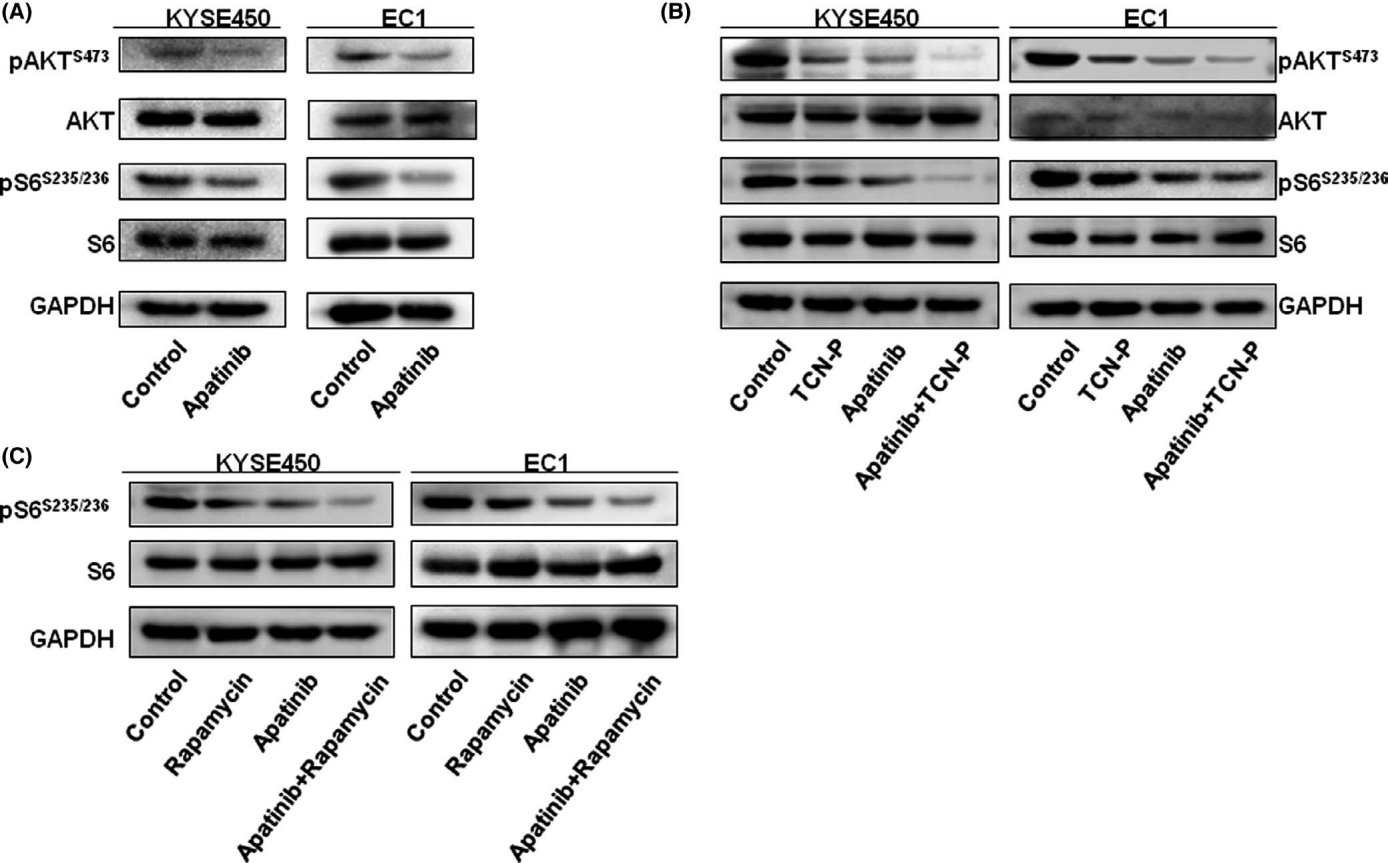
Apatinib reduced activity of Akt/mTOR pathway. (A) Western blot results of Akt, p‐Akt, S6 and p‐S6 protein expression in KYSE450 and EC1 cells treated with apatinib. (B) Western blot results of Akt, p‐Akt, S6 and p‐S6 protein expression in KYSE450 and EC1 cells treated with TCN‐P(Akt pathway inhibitor), apatinib or both agents. (C) Western blot results of S6 and p‐S6 protein expression in KYSE450 and EC1 cells treated with rapamycin (mTOR pathway inhibitor), apatinib or both agents

A relevant research has shown that the activation of Akt/mTOR signalling pathway is vital to tumour development, progression and prognosis in ESCC cells. In this study, we investigated whether apatinib inhibits cell growth of ESCC by blocking Akt/mTOR signalling pathway. Western blotting analysis showed that apatinib inactivated p‐Akt and p‐S6 protein expression in ESCC cells (Figure 5A). To further confirm the results, we, respectively, treated ESCC cells with Akt pathway inhibitor (TCN‐P)/mTOR pathway inhibitor (rapamycin), apatinib and both agents. Western blotting analysis showed that p‐Akt and p‐S6 protein expression was down‐regulated in ESCC cells treated with TCN‐P or apatinib, and apatinib enhanced the effect of Akt pathway inhibitor (Figure 5B). Similarly, apatinib promoted the inhibition influence of the rapamycin (Figure 5C). These results indicated that apatinib suppressed cell growth of ESCC by blocking Akt/mTOR signalling pathway.

### 3.6 Apatinib enhances the inhibition effect of cytotoxic drugs on ESCC cell growth

**FIGURE 6:**
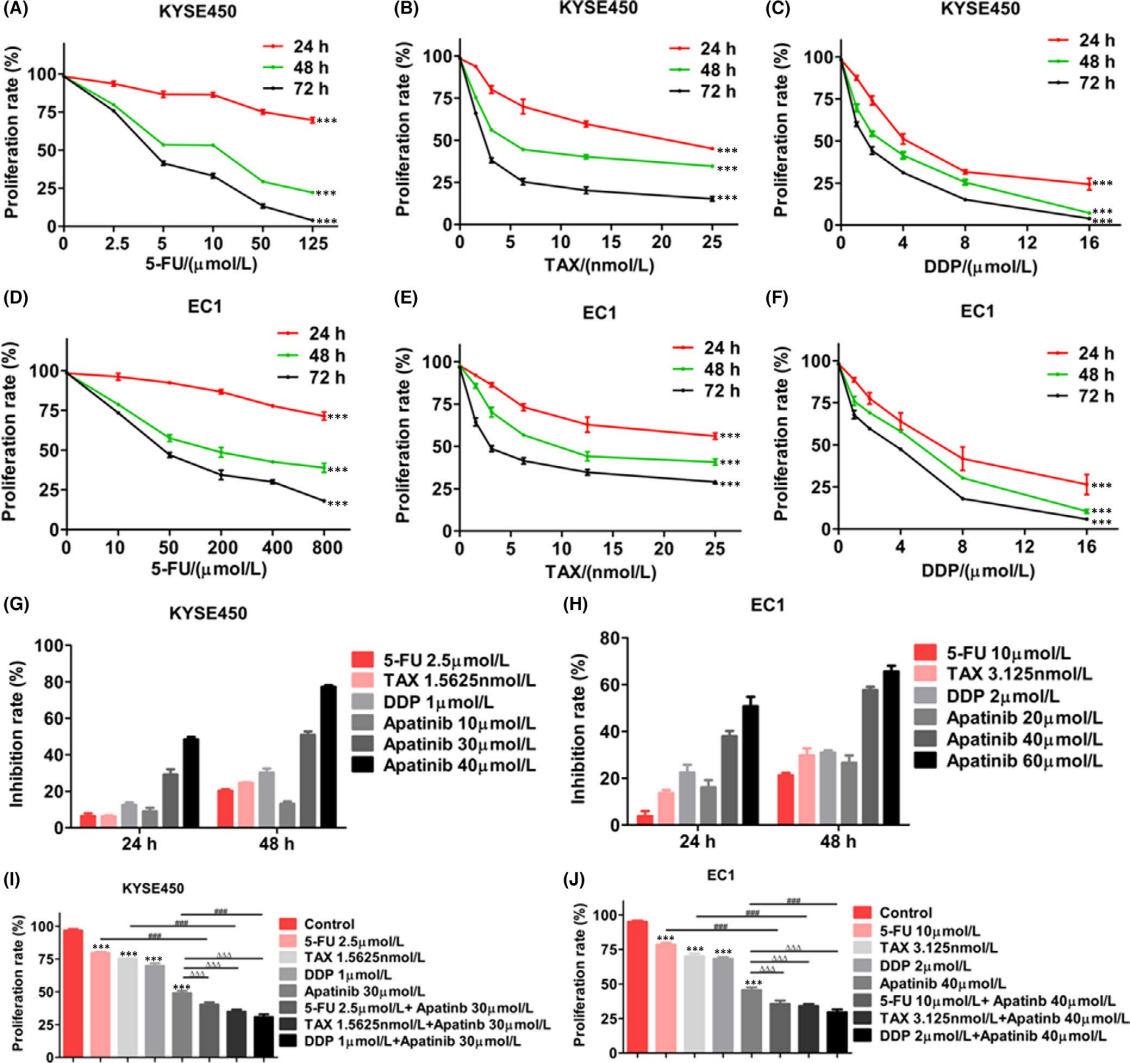
Effects of apatinib combining with cytotoxic drugs on cell proliferation of ESCC cells. (A–C) KYSE450 cells were treated with 5‐Fu or TAX, or DDP at the indicated concentration for 24, 48 and 72 h. Cell viability was determined by CCK‐8 assay. (D–F) EC1 cells were treated with 5‐Fu or TAX, or DDP at the indicated concentration for 24, 48 and 72 h. Cell viability was determined by CCK‐8 assay. (G and H) CCK‐8 results showed apatinib or cytotoxic drugs treatment inhibited cell proliferation of KYSE450 and EC1 cells. (I and J) KYSE450 and EC1 cells were treated with apatinib combining with 5‐Fu or TAX, or DDP for 48 h. Cell viability was determined by CCK‐8 assay. Data are shown as mean ±SEM (compared with control group: *** *p* < 0.001; compared with Apatinib group: ∆∆∆ *p* < 0.001; compared with cytotoxic drugs (TAX, 5‐Fu or DDP): ### *p* < 0.001)

**FIGURE 7:**
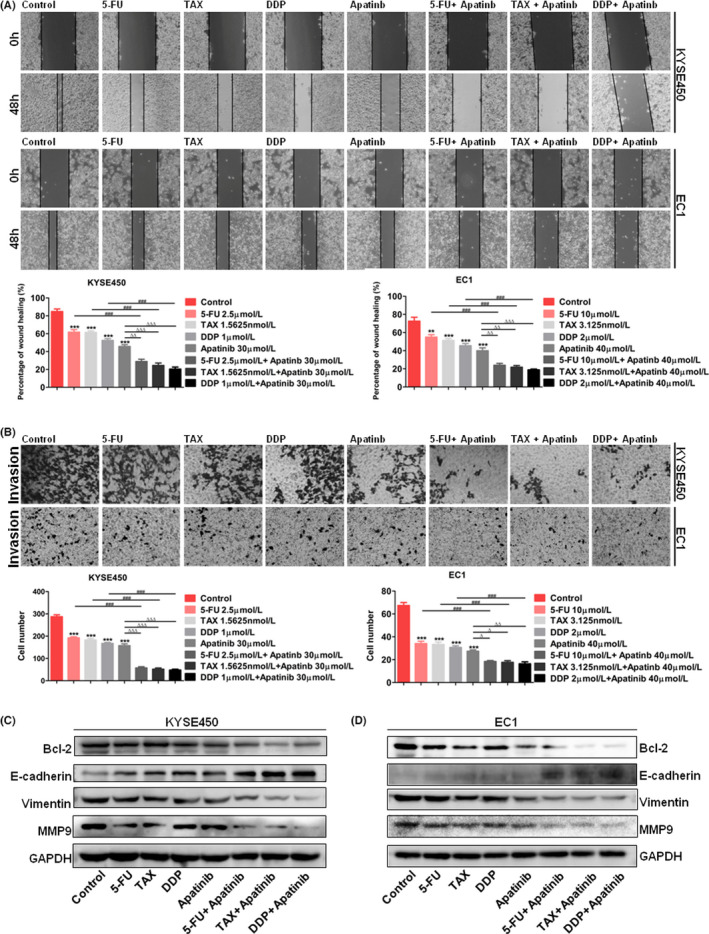
Combination of apatinib with cytotoxic drugs inhibited cell migration and invasion of ESCC. (A) Wound‐healing rates of KYSE450 and EC1 cells treated with apatinib combining with 5‐Fu or TAX, or DDP (×100). (B) Transwell chamber assay showed invasion ability of KYSE450 and EC1 cells treated with apatinib combining with 5‐Fu or TAX, or DDP (×400). (C and D)Western blot analysis of VEGFR‐2, Bcl2, E‐cadherin, vimentin and MMP9 protein expression in KYSE450 and EC1 cells treated with apatinib combining with cisplatin or paclitaxel, or 5‐Fu. Data are shown as mean ± SEM (compared with control group: ** *p* < 0.01, *** *p* < 0.001; compared with Apatinib group: ∆ *p* < 0.05, ∆∆ *p* < 0.01, ∆∆∆ *p* < 0.001; compared with cytotoxic drugs (TAX, 5‐Fu or DDP): ### *p* < 0.001)

KYSE450 and EC1 cells were treated with 5‐Fu, paclitaxel or cisplatin in different concentrations. After 24, 48 and 72 h, proliferation abilities of KYSE450 and EC1 cells were determined by CCK‐8. We found that proliferation rates of cytotoxic drugs in KYSE450 and EC1 cells were lower than those of the untreated control group with the increase of drug dose and treatment time (Figure 6A–F). Proliferation inhibition rates of cytotoxic drugs and Apatinib in ESCC cells at 24 and 48 h were showed in Figure 6G,H. Then, we used apatinib combining with TAX or 5‐FU, or DDP to treat KYSE450 and EC1 cells. The data showed that combination of apatinib with each cytotoxic drug displayed synergistic inhibition effects on the proliferation of KYSE450 and EC1 cells compared with each treatment alone (Figure 6I,J). Western blot analysis showed that combination of apatinib with each cytotoxic drug significantly reduced Bcl2 protein level in KYSE450 and EC1 cells compared with single‐agent treatment groups (Figure 7C,D).

### 3.7 The combination of apatinib with cytotoxic drugs inhibits migration, invasion and EMT of ESCC cells

KYSE450 and EC1 cells were wounded and treated with apatinib combining with 5‐FU or TAX, or DDP. After 48 h, we calculated the healing rates and found that apatinib or cytotoxic drugs alone inhibited the migration of KYSE450 and EC1 cells. Combining apatinib with each cytotoxic drug could more significantly inhibit migration of two cell lines (Figure 7A). After treating KYSE450 and EC1 cells with apatinib and cytotoxic drugs for 24 h, invasion ability of cells was detected by transwell chamber assay. We found that the number of invasive cells treated with apatinib or each cytotoxic drug alone was less than the control group. The number of invasive cells treated with combination of apatinib and each cytotoxic drug was less than single treatment groups (Figure 7B). Western blot analysis showed that the levels of MMP9 and vimentin in oesophageal cancer cells treated with apatinib and cytotoxic drugs were significantly decreased, and E‐cadherin expression was increased (Figure 7C,D).

### 3.8 The combination of apatinib with cytotoxic drugs inhibits the growth of oesophageal tumour in vivo

**FIGURE 8:**
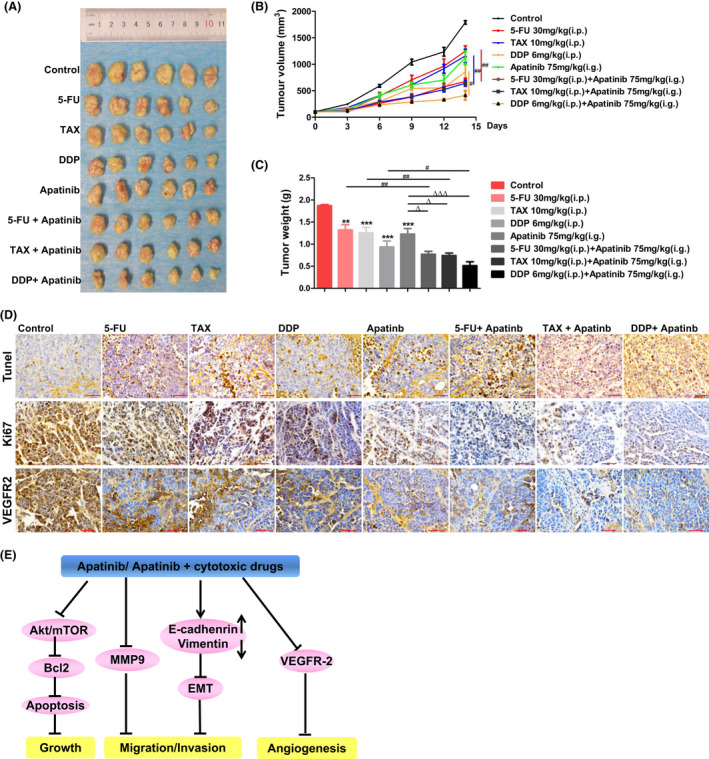
In vivo antitumour effect of apatinib combining with cytotoxic drugs in EC1‐GFP cells bearing nude mice. EC1‐GFP cells were injected into BALB/c nude mice, which were treated with apatinib and cytotoxic drugs according to Table 1 for 14 days. (A) Xenograft tumours were removed from mice after treatment for 14 days. (B) The average of tumour volumes was plotted. (C) Tumour weights of different groups after treatment for 14 days. (D) Tunel and immunohistochemistry of Ki67 and VEGFR‐2 were shown and compared among different groups (×400), bar = 50 μm. (E) A schematic sketch of Apatinib functions in ESCC. Data are shown as mean ± SEM (compared with control group: ** *p* < 0.01, *** *p* < 0.001; compared with Apatinib group: ∆ *p* < 0.05, ∆∆∆ *p* < 0.001; compared with cytotoxic drugs (TAX, 5‐Fu or DDP): # *p* < 0.05, ## *p* < 0.01)

To further access the antitumour function of combination of apatinib and cytotoxic drugs, we used 5‐week‐old female BALB/c nude mice to establish tumour xenografts model. Mice were treated with single agent, combination of apatinib and each cytotoxic drug. The results showed that each agent alone inhibited tumour growth compared with the control group (Figure 8A–C). The combination of apatinib with each cytotoxic drug displayed synergistic inhibition effects on the growth of tumours compared with each treatment alone (Figure 8A–C).

Additionally, Tunel results showed that apoptosis rates of oesophageal tumour increased after each agent treatment compared with control group, which were significantly enhanced in two agent treatment groups compared with each agent treatment groups (Figure 8D).

Immunohistochemistry results showed that the Ki67 protein level was significantly reduced in the combination of apatinib and each cytotoxic drug groups compared with that in each agent groups (Figure 8D). Moreover, the expression levels of VEGFR‐2 in combination of apatinib with each cytotoxic drug groups were lower than that in one agent groups (Figure 8D). These data demonstrated that apatinib markedly suppressed tumour angiogenesis, and the combination of apatinib with each cytotoxic drug had better effect on anti‐angiogenesis in ESCC.

## 4 DISCUSSION

Local recurrence and distant metastasis after surgery are the main death causes in ESCC patients. Tumour angiogenesis plays an important role in tumour growth and metastasis, which is a complex process and predominantly regulated by vascular endothelial growth factor (VEGF) and VEGFR.^20^ Bioinformatic analysis results showed that VEGFR‐2 expression was upregulated in ESCC patients compared with normal. The expression of VEGFR‐2 in advanced stage patients was higher than that in early stage patients. Additionally, high VEGFR‐2 expression was a predictor of poor prognosis for patients with ESCC. Apatinib promotes tumour angiogenesis via binding to VEGF‐A.^21^ Apatinib competitively inhibits the binding of VEGF to VEGFR‐2 and VEGFR‐2 autophosphorylation by binding to VEGFR‐2, which exerts significant antitumour effect.^22^

In our study, the two ESCC patients were found to have distant metastases. Then, the data demonstrated that the combination of apatinib and chemotherapy can control the progression of ESCC. Moreover, a retrospective study showed that apatinib combined with docetaxel can prolong the survival of patients with advanced ECA.^16^ Therefore, apatinib is a potential target drug for ESCC.

Previous studies have reported that apatinib inhibited the progression of several malignancies.^23^, ^24^ Apatinib inhibited cell proliferation, migration and invasion in cancers, such as hepatocellular carcinoma (HCC), colon cancer, osteosarcoma and glioma.^17^, ^18^, ^19^, ^25^, ^26^ To investigate the role of apatinib in ESCC cells, we treated ESCC cells with different density of apatinib. We found that apatinib suppressed cell growth through inducing cell apoptosis in ESCC cells. It was consistent with previous study that apatinib enhanced cell apoptosis by decreasing the expression of Bcl2.^19^, ^25^ Additionally, our data showed that apatinib inhibited migration and invasion of ESCC cells. In addition, it significantly suppressed the expression of metastatic marker MMP9 and mesenchymal marker vimentin, and enhanced the expression of epithelial marker E‐cadherin in ESCC cells. It was consistent with publications that apatinib inhibited cell migration and invasion by blocking the expression of Slug, snail and MMP9 in the cholangiocarcinoma (CCA) cell lines,^27^ and suppressed EMT in osteosarcoma.^17^ These results indicate that apatinib inhibited the progression of malignant neoplasms, including ESCC (Figure 8E).

Akt/mTOR pathway is usually activated in a variety of malignancies. Emerging evidence suggests that Akt/mTOR signalling pathway affect tumour cells function, including cell proliferation, apoptosis, cell cycle, invasion, autophagy and angiogenesis.^25^, ^27^, ^28^ In this study, we confirmed that apatinib inhibited cell growth of ESCC cells through suppressing p‐Akt and p‐S6 expression. Moreover, apatinib enhanced the effects of Akt pathway inhibitor and mTOR pathway inhibitor. It was consistent with previous research in colon cancer and papillary thyroid carcinoma, which showed that apatinib induced cell apoptosis via inactivating Akt/mTOR signalling pathway.^18^, ^29^

Currently, DDP, 5‐FU and TAX are the main recommendation for advanced ESCC first‐line treatment. However, it is difficult to acquire satisfactory clinical efficacy and improve survival time of patients with ESCC. Therefore, more effective treatments are required for clinics to prolong OS of patients with ESCC.

In this research, we chose TAX, 5‐FU and DDP combined with apatinib to investigate whether the inhibition activity increased in ESCC. Compared with each agent treatment groups, the combination of apatinib and cytotoxic drug groups significantly inhibited the growth of ESCC cells in vivo and in vitro. Apatinib combined with single cytotoxic drug promoted cell apoptosis through decreasing the expression of Bcl2. Furthermore, the combination of apatinib with one cytotoxic drug significantly inhibited migration and invasion compared to single agent in ESCC cells. Western blot analysis showed that MMP9 and vimentin protein level in the combination of apatinib with single cytotoxic drug groups was lower than that in one agent treatment groups, and E‐cadherin protein level was higher in two agent treatment groups than that in single‐agent treatment groups. It was consistent with previous reports that apatinib increased the antitumour effect of doxorubicin in side population cells and ABCB1‐overexpressing leukaemia cells.^30^ Furthermore, Tian et al. verified that the synergistic antitumour efficacy of apatinib combining with either docetaxel or doxorubicin in lung cancer xenograft models, oxaliplatin or 5‐FU in colon cancer xenograft models.^2^ Immunohistochemistry results showed that apatinib suppressed angiogenesis through decreasing the expression of CD31.^2^ In our study, Tunel results demonstrated that the number of apoptotic cells of oesophageal tumour in two agent treatment groups was significantly higher than one agent treatment groups. Immunohistochemistry analysis indicated that angiogenesis‐related proteins (VEGFR‐2) expression and prognostic biomarker (Ki67) were inactivated by apatinib combined with cytotoxic drugs compared with single agent. These data indicated that the combination of apatinib and cytotoxic drugs had synergistic antitumour efficacy on ESCC (Figure 8E). However, the mechanism of the synergistic antitumour effect of apatinib combined with chemotherapy drugs on ESCC needed to be further explored.

In summary, we found that the combination of apatinib and a chemotherapeutic drug inhibited the development of ESCC patients. The single agent and the combination of apatinib and each cytotoxic drug enhanced cell apoptosis through inhibiting Bcl2 and repressed cell invasion by blocking MMP9 and EMT in ESCC cells. Moreover, apatinib inhibited cell growth via inactivating Akt/mTOR pathway. Apatinib combined with chemotherapeutic agents inhibited tumour growth by promoting apoptosis, and suppressed angiogenesis via inhibiting VEGFR‐2 and CD31 in vivo.

## CONFLICT OF INTEREST

The authors declare no conflict of interest.

## AUTHOR CONTRIBUTION

**Yanyan Chi:** Conceptualization (equal); Formal analysis (equal); Methodology (equal); Visualization (equal); Writing – original draft (equal); Writing – review & editing (equal). **Feng Wang:** Conceptualization (equal); Supervision (equal); Writing – review & editing (equal). **Yana Zhang:** Methodology (equal). **Zhengzheng Shan:** Methodology (equal). **Weili Tao:** Data curation (equal). **Yujin Lian:** Data curation (equal). **Dao Xin:** Visualization (equal). **Qingxia Fan:** Conceptualization (equal); Resources (equal); Supervision (equal); Writing – review & editing (equal). **Yan Sun:** Conceptualization (equal); Resources (equal); Supervision (equal).

## Data Availability

The data that support the findings of this study are available from the corresponding author upon reasonable request.
